# Case of Supposed Amputation of the Arm of Fœtus in Utero, from Mental Emotion

**Published:** 1869-09

**Authors:** J. L. Kitchen

**Affiliations:** Wyanet, Ill.


### CASE OF SUPPOSED AMPUTATION OF THE ARM OF FŒTUS IN UTERO, FROM MENTAL EMOTION.

WYANET, ILL., *August 15, 1867.*

MR. EDITOR:—Mrs. E. H., of this place, was confined on the 11th of October, 1868, by Dr. P. J. Mulvane. The confinement lasted four hours, and it was observed that the left arm of the child had been amputated at the middle third of the humeral region. Upon investigation, it was ascertained that the mother, shortly after becoming pregnant, visited in the East, and while traveling on the cars witnessed the great toe of a child amputated by the car door shutting upon it violently. The mother attributes the cause of the arm amputation, from the sight of the last mentioned. I have seen the child, which is well and hearty, and since it is a case of unusual interest to me, I thought it might be of sufficient interest for publication.

J. L. KITCHEN, M.D.

